# The Sum and Substance of Antiseptic Surgery

**Published:** 1869-12

**Authors:** E. Andrews

**Affiliations:** Prof. of Principles and Practice of Surgery, Chicago Medical College


ARTICLE XLVI.

### THE SUM AND SUBSTANCE OF ANTISEPTIC SURGERY.

By E. ANDREWS, M.D., Prof. of Principles and Practice of Surgery, Chicago Medical College.

There are many practitioners whose time and opportunities have not enabled them to give carbolic acid, and its kindred agents either a careful trial or full investigation by reading, and who, consequently, do not know how much is truth, and how much is error in what is said on the subject. This article is intended to supply a want by making a condensed statement of this part of surgery. First, then,

WHAT WILL CARBOLIC ACID ACCOMPLISH?

Carbolic acid, creasote, sulphurous acid, permanganate of potash, and all the other poisons, which destroy animalcular life without too much irritation to human tissues, act in the same manner, and produce similar results. Carbolic acid being most used, is, however, here taken as the type.

1. In compound fractures of the most aggravated character, it prevents almost absolutely all suppuration, and most of the soreness and swelling, causing large lacerated openings to go through their process of healing, without any unpleasant smell, and often without the formation of a spoonful of pus.

2. Lacerations and compound fractures laying open large joints, such as the knee, are often healed by it without suppuration, and without exhaustion. Lacerated flesh wounds follow the same law.

3. Lumbar abscesses, and other large collections of pus, opened under a carbolic acid covering, close again by first intention, and after the pus is evacuated, the cavity ceases to produce any more, secreting only a clear bland serum. Meanwhile, the patient, instead of falling into hectic in the usual manner, grows fat and strong, and ultimately recovers.

4. Fistulas communicating with carious bone, may be often healed permanently, without operation, the suppuration ceasing, and the granulations first firmly enclosing the dead bony spicula, and then effecting their removal by absorption. This is, perhaps, the most surprising of the effects of carbolic acid.

5. In surgical operations it may be used to promote union by first intention, and to prevant erysipelas, pyæmia, and other septic forms of disease.

WHAT IT IS.

Carbolic acid (formerly called phenic acid) is a hydrocarbon obtained from coal tar. It can scarcely be termed a true acid, its acid properties being almost nothing. It more properly belongs to the class of alcohols. When pure it crystalizes. It coagulates albumen, and acts as a slight caustic on the tissues, being in that respect rather feebler than nitrate of silver.

DIFFERENT FORMS.

When the crystals are mixed with 5 per cent. of water, they deliquesce and form a permanent fluid. If more water be added it will not mix, but floats on the top, taking up into solution only about 6 per cent. of the acid. If the two solutions are shaken together, they soon separate again, the lower one containing 95 per cent. of the crystalized acid, and the upper about 5 per cent. The upper solution is the one generally to be used for injecting abscesses, etc., the lower one being too strong. There are no officinal names for distinguishing these three forms of the medicine, and hence the manufacturers give them all sorts of titles. The only reliable way is to buy the crystals, and prepare the solutions by adding water.

### THE THEORY OF ITS ACTION.

The atmospheric air always contains, floating as dust in it, a great number of vegetable and animalcular germs, which light upon exposed organic fluids, and multiplying by millions hourly, convert the organic material into a putrifying mass. If the germs be wholly excluded, it is found that putrifaction does not occur, even though warmth and moisture both are present.

Prof. Lister maintains that these putrefacient animalcules are the cause of putridity and irritation, and that the irritation causes the suppuration, and hence, that when no other strong cause of irritation is present, the effusion of pus from any raw tissue may be wholly prevented by simply applying carbolic acid, and killing all the animalcules, and preventing the access of any new ones.

### HOW TO USE IT.

Prof. Lister uses rather complex methods, which can, by no means, be carried out easily by American surgeons, outside of the large cities. During the past year, I have devised various ways of simplifying the applications, with excellent success; and I am glad to see by late European advices that he himself is now resorting to some of the identical modes which I had planned independently.

I have found the following three preparations the most convenient:—First, take about one ounce of the crystals, or of the 95 per cent. solution, which is nearly as strong, and agitate it in a bottle, with 10 or 15 ounces of water; allow it to settle a few minutes, and the clear 5 per cent. solution will appear at the top, and the surplus of acid will settle to the bottom, in the form of the 95 per cent. solution. Also, take about one ounce of the crystals, or of the 95 per cent. solution, and dissolve it in any oil. The best is a pure quality of castor oil, both because it has more viscidity, and is, therefore, better for dressings, and because it will dissolve the 95 per cent. solution of the acid completely, making a perfectly bright, clear compound, which is elegant in its appearance. Other oils precipitate the 5 per cent. of water, in the form of an emulsion, which gives them a dull look. Finally, take eight parts of collodion, and mix them with one part of carbolic acid. We are now armed with three preparations, *viz.:* the carbolated water (5 per cent. solution), the carbolated oil, and the carbolated collodion, and are prepared for action.

TREATMENT OF COMPOUND FRACTURES.

The fracture being adjusted and splints applied, the wound should be washed to its remotest corner, with the 5 per cent. solution, using a syringe, if necessary, to throw it fully into deep parts. This will kill all the animalcular germs present in the wound. The next step is to prevent the admission of any more. For this purpose, take a pledget of lint or cotton batting, large enough to fill the wound, if it is open, or to more than cover the orifice if closed, and lay it in or on, as the case may be. If elegance is sought after, a piece of tinfoil or oiled silk may be placed over the lint, to prevent the oil from soiling the outside dressings. Finally, confine the whole in place with a roller, or with a Mayor's handkerchief. The next day, the injection and dressing must be gently repeated. It should be observed, that new wounds and freshly opened abscesses, whose raw nerves are not yet covered with granulations, often feel the smart of the first wash severely. If so, the second wash may be reduced to one-third strength, gradually increasing again to 5 per cent., or 20 grs. to the ounce. Under this treatment, frequently there is not an ounce of pus secreted during the whole cure, and the bone often unites as promptly as in a simple fracture, an important gain, as all know how prone compound fractures are to slow union.

LUMBAR AND OTHER LARGE ABSCESSES.

These large collections of pus, which often destroy the patient soon after they are opened, may generally be rendered surprisingly harmless in the following manner:—Dip a trochar in carbolated water, and thrust it obliquely into the abscess, so as to make a valvular opening. Let the pus flow as long as it will, or press it gently out, but do not allow a particle of air to regurgitate through the canula, as that would introduce animalcular germs. When the pus ceases to flow, dip a few fibers of cotton batting into the carbolated collodion, lay them over the point of entrance to the trochar, then placing the thumb on the end of the canula, or a cork in it, to prevent any regurgitation of air, draw it out from under the collodionized cotton pressing, the latter, instantly, upon the orifice, to prevent the admission of living germs. In this way, the orifice will be sealed, and a union, by first intention, usually obtained. After a week or more, the abscess will generally be found filled again, when it should be again tapped in the same way. The pus will now be found thinner and more serous. After a longer interval, a third tapping will find the pus almost transparent, and the fourth one often will draw absolutely transparent serum. In this way, the intervals will lengthen, and the abscess, at least, in many instances, be gradually made to wither away and disappear, the patient all the while growing fat and rosy, and presenting a total contrast to the usual results of a lumbar abscess. Sometimes the abscess is clogged with masses of dead areolar tissue, so that it is impossible to evacuate it through the canula. In this case, it may be still possible to draw them out through the tube by slender forceps, dipped before each insertion in carbolated water, or oil; but if this fails, or if the collodion does not produce a union by first intention, or if it has been already opened before coming under treatment, a different method is necessary. We must now proceed by injections. For this purpose, use a 1 or 2 per cent. solution, the first few days, gradually increasing to five per cent., or 20 grs. to the ounce, as the patient becomes used to it. This must be thrown in once a day, filling the abscess completely. Then allowing the solution to run out, a large pledget of cotton, dipped in carbolated oil, must be placed over the fistula, and confined by a bandage. It will be the neater and better if a piece of druggist's tin-foil is placed between the cotton and the bandage. This dressing must be repeated daily. Frequently the flow of pus will continue, gradually diminishing for one, two, or three weeks under this treatment; but it will be almost always brought under control, and the abscess at length discharge only a little transparent serum, without any odor. This may continue many months, but the patient is saved from hectic, grows fat and vigorous, and his life is commonly saved.

CARIOUS JOINTS.

Prof. Lister claims that carbolic acid will heal up a fistula over necrosed bone, and cause its absorption. In my own practice, I have been able to do this, where the sequestrum was of cancellar tissue, or a fragment of compact tissue, of small size; but large fragments of compact bone, two or three inches long, have, in my hands, resisted the treatment, and required operation. The principle appears to be this:—Cancellar bone, if suppuration be suppressed by the injections, becomes permeated with the granulations which hug close to all the little spiculæ, and being in absolute contact, effect their absorption; but large compact fragments cannot be thus interpenetrated with living granulations, and hence their absorption, if accomplished at all, would, probably, be too slow to be waited for. At any rate, such is my experience up to the present time. The smaller joints do admirably under the daily carbolated injections and dressings. I have repeatedly healed them up in a complete and permanent manner, after the probe showed the articular surfaces of the bones to be thoroughly carious. Anchylosis, of course, occurs. Finger-joints may be often healed in two weeks, but larger ones require much more time. I have an elbow whose articular surfaces were perfectly carious, six months ago; the limb was swollen and red, discharging large quantities of stinking pus, and the patient was emaciated, cadaverous, and hectic. Under the antiseptic dressings, he has grown plump and vigorous, the swelling has all disappeared, and the skin resumed its natural whiteness. For three months, I have been unable to find any dead bone with the probe, and there is no discharge of pus, yet the fistulas are still permeable to the probe, and probably will not close under several months more.

Wounded and carious knee-joints can often be treated with splendid success in the same way, compared with the disasters which used to follow these conditions. I have one now on hand which does not discharge a teaspoonful of pus in a week, and the patient will save both his life and his limb. I have seen one compound fracture, penetrating the knee, thus treated, where the joint did not even inflame, and was not anchylosed. Some carious knees, however, are too extensively diseased to admit of final cure, and the antiseptic treatment is only useful to arrest exhaustion, and enable the surgeon to get them up to a condition vigorous enough to bear an operation. The abscesses of hip-disease often respond admirably to the same treatment.

### INCISED WOUNDS.

Wash the wound thoroughly to its remotest recesses with the carbolated water, then close it with sutures, and add adhesive straps or carbolated collodion. If the straps are used, a pledget of lint, dipped in carbolated oil, should be laid over the wound and covered with tin-foil and bandages. In this way, union by first intention is greatly promoted, and the risk of erysipelas almost absolutely annihilated.

If any one, however, supposes that carbolic acid is infallible, he will be disappointed. Many particular cases occur where it partly fails of its end. In these instances, it will often be found that a solution of one part of the officinal sulphurious acid to two parts of water does better. Solutions of permanganate of potash, two grains to the ounce, sometimes do well; and, in the same manner, other antiseptic solutions, if used in sufficient strength, occasionally succeed. Many persons miss entirely the effects of the antiseptic treatment, because they are inefficient in its application. If they do not effectually destroy every animalcule in the abscess or wound, and absolutely bar out the access of every new germ in the living state, they accomplish nothing. The washing and injection must be thorough, and the carbolated outside dressings must cover the whole orifice and its vicinity. When these principles are observed, the success is wonderful.

On the whole, it must be acknowledged that the use of carbolic acid has revolutionized certain branches of surgery, and enables us to save many limbs and lives which would formerly have been lost. I would advise no one to make a hobby of it, but that it is a remarkable addition to the resources of our art is a fixed and undeniable fact.

